# Beyond a Name Change: Polyendocrine Metabolic Ovarian Syndrome and a New Era of Opportunity for Research, Care, and Advocacy

**DOI:** 10.5812/ijem-172704

**Published:** 2026-06-29

**Authors:** Fahimeh Ramezani Tehrani, Fereidoun Azizi, Samira Behboudi-Gandevani

**Affiliations:** 1 Reproductive Endocrinology Research Center, Research Institute for Endocrine Molecular Biology, Research Institute for Endocrine Sciences, Shahid Beheshti University of Medical Sciences, Tehran, Iran; 2 Foundation for Research and Education Excellence, Vestavia Hills, United States; 3 Endocrine Research Center, Research Institute for Endocrine Disorders, Research Institute for Endocrine Sciences, Shahid Beheshti University of Medical Sciences, Tehran, Iran; 4 Faculty of Nursing and Health Sciences, Nord University, Bodø, Norway

**Keywords:** Polycystic Ovarian Metabolic Syndrome, New Era, Advocacy

Polycystic ovary syndrome (PCOS) is a highly prevalent endocrine-metabolic disorder, affecting 11 - 18% of women of reproductive age in Iran (1). Beyond its reproductive features, PCOS is increasingly recognized as a systemic condition that predisposes women to a spectrum of cardiometabolic abnormalities (2). In May 2026, following more than a decade of international collaboration involving more than 22,000 patients, clinicians, researchers, and advocacy organizations across six continents, PCOS was formally renamed polyendocrine metabolic ovarian syndrome (PMOS) (3). This landmark development represents more than a terminological revision; it reflects a fundamental reappraisal of one of the most common yet persistently misunderstood conditions in women’s health, affecting approximately 1 in 8 women worldwide (4). The adoption of PMOS signals an important shift in how the condition is conceptualized, investigated, and managed.

For decades, the term polycystic ovary syndrome has been criticized as scientifically inaccurate and clinically limiting. The name places undue emphasis on ovarian morphology, although ovarian cysts are neither required for diagnosis nor representative of the syndrome’s underlying biology. More importantly, PCOS is now recognized as a complex endocrine-metabolic disorder with reproductive, metabolic, psychological, and long-term cardiometabolic consequences (5). Beyond ovulatory dysfunction and infertility, affected individuals face increased risks of insulin resistance, obesity, type 2 diabetes, cardiovascular disease, mental health disorders, and impaired quality of life (6). Traditional terminology has failed to capture this complexity and may inadvertently contribute to under-recognition of key aspects of care.

The significance of this change extends beyond terminology. Disease names influence scientific inquiry, clinical practice, healthcare policy, and public perception. Historically, the ovarian-centric framing of PCOS has focused attention primarily on reproductive manifestations, whereas metabolic abnormalities and psychological comorbidities have often received less emphasis, despite their substantial contribution to long-term morbidity. By explicitly incorporating the terms polyendocrine and metabolic, PMOS more accurately reflects the contemporary understanding of the disorder and acknowledges the complex interactions among androgen excess, insulin resistance, neuroendocrine dysfunction, adipose tissue biology, inflammation, and ovarian dysfunction. Importantly, the new terminology recognizes PMOS as a lifelong, multisystem condition requiring multidisciplinary management and long-term surveillance, rather than as a disorder confined to reproductive health.

The adoption of PMOS also creates important opportunities for research. For many years, funding priorities and investigative efforts have disproportionately focused on fertility-related outcomes. Although reproductive health remains a critical component of the syndrome, future research agendas can more effectively address its interconnected endocrine, metabolic, cardiovascular, dermatological, and psychological dimensions. The new nomenclature may strengthen recognition of PMOS as a major noncommunicable disease with significant public health implications, thereby supporting greater investment in mechanistic, translational, and population-based research.

From a clinical perspective, renaming the condition may encourage a more holistic approach to care. When endocrine, metabolic, and ovarian features are reflected in the nomenclature itself, healthcare professionals may be more likely to recognize the full spectrum of disease manifestations. Such a shift could facilitate earlier diagnosis, multidisciplinary care pathways, and more effective prevention of long-term complications. This approach is consistent with growing evidence that PMOS should be managed as a lifelong condition extending well beyond the reproductive years.

The implications for therapeutic innovation are equally noteworthy. Recognition of the metabolic nature of PMOS may accelerate investigation of interventions targeting insulin resistance, obesity, inflammation, and cardiometabolic risk, moving beyond symptom-based treatment toward mechanism-based interventions.

Another important consequence relates to patient empowerment. Notably, the consensus process incorporated patient perspectives throughout its development, reflecting a genuinely patient-centered approach. Individuals living with the condition consistently reported that the term PCOS failed to reflect their lived experiences and often minimized the seriousness of their symptoms (7). By acknowledging the systemic nature of the disorder, PMOS may improve patient understanding, reduce stigma, and validate experiences that have historically been overlooked. Furthermore, more accurate terminology may strengthen advocacy efforts, enhance public awareness, support research funding initiatives, and help policymakers recognize the substantial health burden associated with the condition.

Nevertheless, challenges remain. A name change alone will not address longstanding problems of underdiagnosis, delayed diagnosis, inadequate professional education, or limited treatment options. Successful implementation will require coordinated updates to clinical guidelines, medical curricula, electronic health records, patient education materials, and disease classification systems, including the International Classification of Diseases. During the proposed transition period, a dual-labeling approach, “PMOS (formerly PCOS),” will be essential to preserve continuity in clinical practice and research.

It is also important to recognize that the diagnostic criteria were not revised as part of the renaming process. The Rotterdam criteria remain unchanged, and whether future diagnostic frameworks will more explicitly incorporate metabolic and neuroendocrine features remains an important question for the field. The nomenclature change may therefore represent not the conclusion of a process, but the beginning of a broader re-evaluation of how this heterogeneous condition is defined and classified.

The transition from PCOS to PMOS offers an opportunity to align nomenclature with contemporary scientific evidence and patient experience. More importantly, it provides a platform to redefine how clinicians, researchers, policymakers, and society understand this highly prevalent condition. If accompanied by meaningful advances in research priorities, healthcare delivery, and public policy, the adoption of PMOS may catalyze earlier diagnosis, more comprehensive care, greater research investment, and improved health outcomes for the millions of women affected worldwide.
